# Aesthetic quality of psychedelic experience is linked to insight and psychological outcomes

**DOI:** 10.3389/fpsyg.2025.1533055

**Published:** 2025-05-15

**Authors:** Jake F. Hooper, Emily L. Gyongyosi, Kent E. Hutchison, Raeghan L. Mueller

**Affiliations:** Department of Psychiatry, University of Colorado, Anschutz Medical Campus, Aurora, CO, United States

**Keywords:** aesthetic, psychedelic, insight, perception, emotion

## Abstract

**Introduction:**

The aesthetic qualities of psychedelic experiences have long been documented, but their specific contribution to therapeutic outcomes remains understudied. Aesthetic experiences may facilitate emotional breakthroughs and cognitive shifts, potentially playing a crucial role in the lasting psychological benefits observed following psychedelic use.

**Methods:**

This cross-sectional study surveyed 96 individuals who reported using classic psychedelics (e.g., psilocybin, LSD, DMT, mescaline) within the past year. Participants completed validated measures including the Mystical Experience Questionnaire (MEQ), Emotional Breakthrough Inventory (EBI), Psychological Insight Scale (PIS), Challenging Experience Questionnaire (CEQ), and a novel measure of aesthetic experience (PAEQ). Linear regression and Spearman correlations were used to assess associations between aesthetic experience and psychological outcomes.

**Results:**

Aesthetic experience was significantly associated with greater emotional breakthrough (*r* = .40), psychological insight (*r* = .48), behavioral change (*r* = .55), and mystical experience (*r* = .49) (all *p* < .001). Aesthetic experience was also inversely associated with fear (*r* = –.24, *p* < .05) and paranoia (*r* = –.36, *p* < .001). Regression models indicated aesthetic quality predicted improvements in depression, anxiety, and quality of life, independent of age, gender, or mystical experience.

**Discussion:**

These findings suggest that aesthetic aspects of psychedelic experiences are not merely perceptual enhancements but may actively contribute to therapeutic outcomes. The inverse relationship between aesthetic quality and challenging experiences highlights the potential utility of optimizing the aesthetic environment in psychedelic-assisted therapy. Future research should further validate the PAEQ and explore causal mechanisms using longitudinal and experimental designs.

## Introduction

Classic psychedelics, such as psilocybin, mescaline, LSD, and DMT, profoundly impact perception, cognition, and emotion, often resulting in deeply meaningful and transformative experiences (Griffiths et al., 2006; Nichols, 2016). Several studies have shown that classic psychedelics are effective for treating a range of mood and substance use disorders under supportive conditions (Griffiths et al., 2016; Davis et al., 2021, 2023; Bogenschutz et al., 2022). Evidence indicates that these therapeutic effects can be long-lasting, providing sustained clinical relief for months or even years (Aday et al., 2020; Agin-Liebes et al., 2020). Agonism at 5-HT2A receptors is thought to be primarily responsible for their subjective effects (Preller et al., 2017; Stenbæk et al., 2021), although the mechanism of action in clinical studies is not fully understood. While neurobiological mechanisms are undoubtedly crucial, they alone may not fully account for enduring beneficial effects (Yaden and Griffiths, 2021). The effects of psychedelics are highly dependent on contextual variables, including the environment and mindset of the individual, often referred to as “set and setting” (Eisner, 1997; Hartogsohn, 2016; Carhart-Harris et al., 2018). This sensitivity to context suggests that the psychological and experiential dimensions play a critical role in shaping the outcomes of the psychedelic experience.

One such factor that remains understudied is the aesthetic quality of the psychedelic experience. The aesthetic experience is a complex emergent phenomenon that occurs as a result of the dynamic interplay between sensory perception, emotional valuation, and cognitive interpretation, mediated by distributed neural networks, including the default mode network (DMN), salience network, and visual processing regions, which together shape meaning-making, psychological insight, and subjective appreciation of beauty (Chatterjee and Vartanian, 2014; Pearce et al., 2016; Belfi et al., 2019). Aesthetic experiences play a significant role in evoking emotional responses and facilitating shifts in cognition and behavior (Mastandrea et al., 2019). Psychedelic substances are well-documented for their ability to enhance sensory and emotional sensitivity, leading to characteristic hallucinations and deeply felt emotional responses. Some of these responses can be seen in psychedelic art, indicating that psychedelics might possess their own aesthetic (Krippner, 2017). Induced aesthetic enhancements—ranging from intensified colors and patterns to the perception of symbolic imagery—engage distributed neural networks related to perceptual and semantic regulation, such as the DMN, and may facilitate emotional breakthroughs (Carhart-Harris et al., 2012; Kaelen et al., 2015; Pasquini et al., 2020). Changes in sensory, cognitive, and emotional processes during altered states contribute to profound psychological shifts, often resulting in lasting therapeutic outcomes, such as greater psychological flexibility and reduced symptoms of anxiety and depression (Roseman et al., 2019; Barrett et al., 2020). However, it remains unclear whether the perceived aesthetic quality of these experiences directly contributes to the positive psychological outcomes frequently reported by recreational users and in clinical studies.

Beyond their impact on individual perception, aesthetic experiences induced by psychedelics may play a role in broader cognitive and existential frameworks, shaping how individuals interpret and integrate their experiences. Previous research has linked peak experiences—including those marked by intense beauty, awe, and transcendence—to long-term shifts in personality traits such as openness and well-being (MacLean et al., 2011; Majić et al., 2015). Similarly, profound aesthetic alterations reported under psychedelics might function as a catalyst for meaning-making, where visually and emotionally salient stimuli trigger insight, emotional breakthroughs, metaphysical beliefs, and an expanded sense of self (Kettner et al., 2021; Timmermann et al., 2021). Thus, aesthetic perception under altered states of consciousness could represent an important mechanism through which psychedelics facilitate broad psychological change.

Given this, the current study aimed to explore how aesthetic qualities influence both positive and negative aspects of the psychedelic experience, particularly those linked to long-term outcomes for depression and anxiety (Peill et al., 2022). Using a web-based survey, participants reported exclusively on their most ‘typical’ psychedelic experience, evaluating aesthetic quality, positive emotions (e.g., emotional breakthroughs, psychological insight), negative emotions (e.g., distress, fear, paranoia), and related outcomes such as changes in depression and anxiety. We hypothesized that higher aesthetic pleasure would be associated with positive psychological outcomes, while negative aesthetic experiences would be associated with challenging experiences and worse psychological outcomes. We examine the connection between psychedelic-induced aesthetics and emotional responses and aim to contribute to the understanding of how psychedelics generate lasting psychological benefits, with potential implications for therapeutic applications.

## Materials and methods

### Design and procedures

We conducted a voluntary and anonymous online survey from July to September of 2024 to gather data on the attitudes and experiences of U.S. adults (18 and older) who do or do not use psychedelic substances. The survey, hosted on the Research Electronic Data Capture platform (REDCap, www.redcap.com), focused on demographics, health, and substance use. The University of Colorado Multiple Institutional Review Board approved the study, and participants provided informed consent by signing the consent form electronically.

Participants were recruited through various channels, including social media platforms, word-of-mouth, in-person outreach, and snowball sampling (i.e., referrals within social networks). The primary recruitment effort targeted participants through advertisements on Meta and Google, specifically from individuals residing in Colorado. These materials directed participants to the online survey form. Individuals under the age of 18 were excluded prior to the survey. No additional criteria were imposed for enrollment. Eligible participants were presented with an informed consent sheet outlining the study’s purpose, risks, benefits, and contact information for the research team. Upon consenting, participants were routed directly to the survey questions. At the conclusion of the survey, participants were debriefed and provided the option to enter an email into a drawing for a chance to win a $100 Amazon gift card. The following analysis used only complete data from participants who indicated current (within one year) classical psychedelic use.

### Self-report measures

#### Assessment of positive experiences

The *Emotional Breakthrough Inventory* (EBI) (Roseman et al., 2019) measured the intensity of emotional breakthroughs during respondents’ typical psychedelic experience, capturing elements such as emotional release, confronting challenging emotions, and gaining insights into emotional issues. The EBI includes 6 items (*α* = 0.94), each rated on a scale from 0 (disagree) to 100 (agree), where higher scores reflect a more significant emotional breakthrough. When used in combination with the CEQ and MEQ, Roseman et al. (2019) found that these measures, when integrated, provide a more comprehensive assessment of psychedelic experiences and significantly enhance the prediction of changes in well-being by capturing a wider range of multidimensional aspects.

The *Mystical Experience Questionnaire* (MEQ) (Barrett et al., 2015; MacLean et al., 2012) measured the intensity of respondents’ typical psychedelic experiences, focusing on mystical characteristics. It comprises 30 items grouped into four dimensions: mystical, positive mood, transcendence of time and space, and ineffability. Each item is rated on a 6-point scale from 0 (none) to 5 (extreme), with higher scores indicating a more intense mystical experience across each dimension. The MEQ has been validated through factor analysis of psilocybin-related experiences (MacLean et al., 2012) and has consistently been associated with long-lasting positive outcomes in both healthy and clinical populations following psychedelic induced peak experiences (Garcia-Romeu et al., 2019; Roseman et al., 2019).

The *Psychological Insight Scale* (PIS) (Peill et al., 2022) is a 6-item (*α* = 0.94) measure used to evaluate the degree of psychological insight gained following respondents’ typical psychedelic experience. Respondents are asked to indicate their level of agreement from 1 (strongly disagree) to 7 (strongly agree) with statements about their psychological insights and changes in behavior following a psychedelic experience. Averaged raw scores generate a total PIS Average score, with higher scores reflecting greater levels of insight and a higher likelihood of meaningful psychological or behavioral change. Post-experience psychological outcomes were evaluated using the PIS-6 average score and the single item PIS-7 on behavioral change. Insight is shown to correlate with decreased symptoms of depression and anxiety (Davis et al., 2020) and mediate the relationship between emotional breakthrough and long-term well-being (Peill et al., 2022). In combination with EBI, CEQ, and MEQ, the PIS is a good predictor of long-term psychological well-being.

#### Assessment of negative experiences

The *Challenging Experience Questionnaire* (CEQ) (Barrett et al., 2016) is a 26-item measure that evaluated the intensity of difficult and distressing emotions experienced during respondents’ typical psychedelic experience. Each item is rated from 0 (none) to 5 (extreme), and the total score is derived by averaging responses across the seven dimensions as a percent: fear, grief, paranoia, isolation, insanity, death and physical distress. The CEQ is often negatively correlated with measures of psychological insight and well-being, highlighting its role in identifying potential psychological risks during psychedelic use. Higher scores on the CEQ indicate more intense and frequent challenging experiences.

#### Assessment of aesthetic experience

The *Psychedelic Aesthetic Experience Questionnaire* (PAEQ) assessed the aesthetic aspects of respondents’ typical psychedelic experiences, drawing upon the conceptual framework of the Aesthetic Experience Questionnaire (AEQ) (Wanzer et al., 2020) and the aesthetic triad model (Chatterjee and Vartanian, 2014), which evaluates aesthetic experiences in terms of sensory, affective, and semantic dimensions. The single-item version (PAEQ-S) aims to capture the overall aesthetic pleasure of the experience through a single item: *“The experience was aesthetically pleasing,*” rated on an 8-point scale from 0 (strongly disagree) to 7 (strongly agree). The single-item measure provides a broad assessment of the aesthetic experience as a whole, making it suitable for exploratory analyses requiring concise self-report data. In contrast, the full 21-item PAEQ provides a more detailed and nuanced evaluation by assessing specific aspects of the aesthetic experience across its intended dimensions. The full 21-item PAEQ is provided in the Supplementary materials, although it was excluded from the present analysis as it has not yet been validated. Future research should focus on refining and validating the scale and its factor structure to more thoroughly explore the associations examined in this exploratory analysis.

#### Assessment of outcomes

Both behavioral and psychological outcomes were assessed using a questionnaire that referred to any change caused by the specific classic psychedelic(s) a participant reported using in their typical experience in the past year (psilocybin, LSD, DMT, or mescaline). The outcomes measured included anxiety, depression, pain, sleep, opioid use, alcohol use, and quality of life. These items were evaluated on a 7-point Likert scale, ranging from −3 (much worse) to 3 (much improved). Each outcome was standardized across substances and, when applicable, aggregated per individual to generate an average outcome value, ensuring the results remained generalized across substances. This questionnaire always referred to the individual’s typical psychedelic experience while accounting for the specific substances used.

### Statistical analysis

To assess the relationship aesthetic experience has to positive and negative experience and outcomes, both Spearman’s rank correlation coefficient and linear regression analyses were conducted. Spearman’s correlation was employed to assess bivariate relationships due to its robustness against non-normally distributed data and ability to detect monotonic associations. Linear regression models were used to further evaluate the predictive nature of these relationships while controlling for potential confounders such as age and gender. Assumptions for the regression models, including linearity, homoscedasticity, and normality of residuals, were checked and found to be adequately met. Multicollinearity was not an issue, as none of the predictor variables showed correlations greater than 0.7. Effect sizes for correlations were expressed as Cohen’s *d*, interpreted as follows: ≥0.2 to <0.44 (small), ≥0.45 to <0.8 (medium), and ≥0.8 (large) (Lakens, 2013). Bootstrapping was performed to compute 95% confidence intervals. Data processing and analysis of outcomes were conducted in the R statistical package (R Core Team, 2022). Analyses of acute positive and negative experiences were conducted using IBM SPSS Statistics version 29.

## Results

### Sample characteristics

Ninety six survey respondents were included in the present analysis (mean age = 40.1, standard deviation (SD) = 13.2). The study cohort was predominantly male (~50%), with 79.2% identifying as white and 77.1% as non-Hispanic. More than half of the participants (56.2%) had completed an associate’s degree or higher, while 54.2% were employed full-time. Regarding income, 34.4% reported earning between $50,000 and $100,000 annually. Regarding substance use, the majority of participants (70.8%) reported having used psilocybin, followed by LSD (26%), DMT (12.5%), and mescaline (1%). Additionally, 17.7% of participants reported using two or more different psychedelics during their typical experience, describing a notable subset of polydrug psychedelic users. These patterns suggest that psilocybin was the most commonly experienced psychedelic among respondents, aligning with broader trends in psychedelic research and accessibility. See Table 1 for additional information on demographics and sample characteristics. See Table 2 for descriptive characteristics.

**Table 1:** Sociodemographic characteristics of participants.

Characteristic	*n*	*%*
Gender (% Female)	36	37.5
Ethnicity (% Hispanic)	20	20.8
Race (% White)	76	79.2
Employment (% Full Time)	52	54.2
Full-time	52	54.2
Part-time	15	15.6
Stay at home parent	7	7.3
Student	3	3.1
Unemployed	11	11.5
Retired	8	8.3
Education		
Less than high school	1	1
High school diploma or GED	18	18.8
Some college	23	24
Associate’s degree	20	20.8
Bachelor’s degree	24	25
Master’s degree	8	8.3
Doctoral degree	2	2.1
Income		
0-50 k	39	40.6
50 k-100 k	33	34.4
100 k+	24	24.8
Substance		
LSD	25	26
Psilocybin	68	70.8
DMT	12	12.5
Mescaline	1	1

**Table 2:** Descriptive statistics.

Variable	Mean	SD	Skew
Age	40.14	13.12	−1.50
Aesthetic experience (PAEQ-S)	5.55	1.82	−1.50
Emotional breakthrough (EBI)	70.26	22.10	−0.99
Psychological insight (PIS-6)	74.59	21.73	−1.41
Behavioral change (PIS-7)	74.97	24.58	−1.40
Mystical experience (MEQ)	2.99	1.19	−0.64
Mystical^a^	3.02	1.40	−0.49
Positive mood^a^	3.44	1.24	−1.19
Transcendence^a^	4.83	1.29	−0.12
Ineffability^a^	3.53	1.33	−1.10
Challenging experience (CEQ)	5.76	7.27	2.93
Fear^b^	5.08	8.39	3.00
Grief^b^	7.40	8.62	1.66
Physical distress^b^	7.21	8.44	1.94
Insanity^b^	4.58	9.17	3.22
Isolation^b^	5.31	9.03	2.35
Death^b^	4.64	9.69	2.79
Paranoia^b^	2.50	7.22	3.76

^a^MEQ subscale. ^b^CEQ subscale.

### Association of aesthetic experience with positive experiences

**Figure 1:**
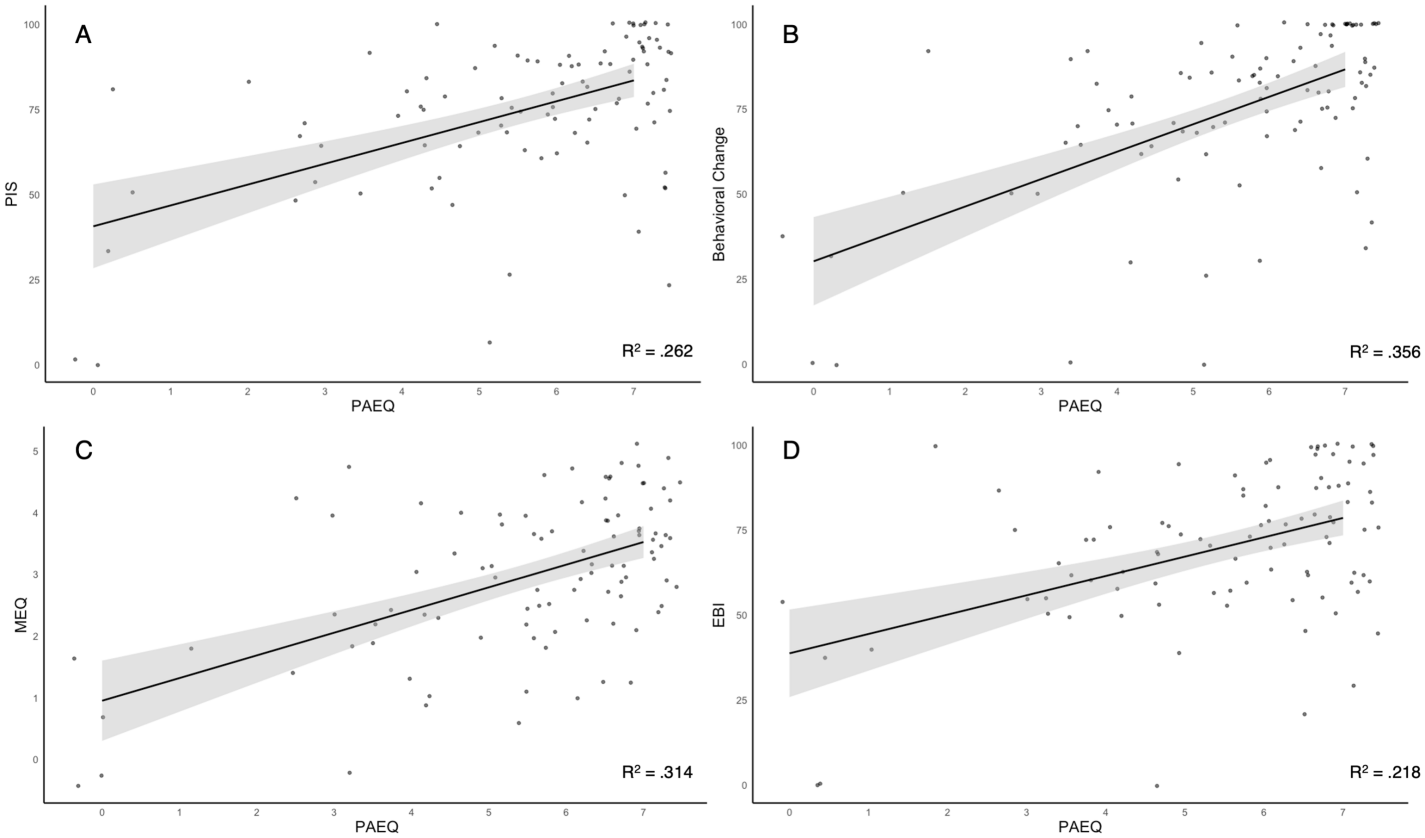
Scatterplots between PAEQ single-item and PIS-6 average **(A)**, behavioral change **(B)**, MEQ Total **(C)**, and EBI average **(D)** variables describing significant moderate positive correlations (*r* = 0.477***, *r* = 0.548***, *r* = 0.398***, *r* = 0.489***). Jitter was added to improve visual clarity and was not used to transform the data. Linear regression lines with 95% confidence intervals are overlaid in grey. Correlation coefficients of determination (R^2^) are reported; ***p < 0.001.

Spearman correlations and regression analysis were performed to determine the relationship between aesthetic experience and positive psychological outcomes. Significant positive correlations were found between aesthetic experience and several positive psychological outcomes (see Figure 1). Aesthetic experience was moderately correlated with emotional breakthroughs (EBI) (*r*(94) = 0.40, *p* < 0.001), psychological insight (PIS-6) (*r*(94) = 0.48, *p* < 0.001), positive behavior change (PIS-7) (*r*(94) = 0.55, *p* < 0.001), and mystical experiences (MEQ) (*r*(94) = 0.49, *p* < 0.001). See Table 3 for a correlation matrix.

**Table 3:** Spearman correlations.

Variable	1	2	3	4	5	6	7	8	9	10	11	12	13	14	15	16
1. Aesthetic Experience	–	–	–	–	–	–	–	–	–	–	–	–	–	–	–	–
2. EBI Average	0.398**	–	–	–	–	–	–	–	–	–	–	–	–	–	–	–
3. PIS-6 Average	0.477**	0.702**	–	–	–	–	–	–	–	–	–	–	–	–	–	–
4. PIS-7	0.548**	0.712**	0.746**	–	–	–	–	–	–	–	–	–	–	–	–	–
5. MEQ Total	0.489**	0.541**	0.559**	0.525**	–	–	–	–	–	–	–	–	–	–	–	–
6. MEQ Mystical	0.383**	0.534**	0.506**	0.490**	0.930**	–	–	–	–	–	–	–	–	–	–	–
7. MEQ Positive	0.641**	0.433**	0.464**	0.499**	0.771**	0.614**	–	–	–	–	–	–	–	–	–	–
8. MEQ Transcendence	0.319**	0.384**	0.421**	0.323**	0.866**	0.782**	0.586**	–	–	–	–	–	–	–	–	–
9. MEQ Ineffability	0.439**	0.348**	0.382**	0.410**	0.598**	0.436**	0.518**	0.514**	–	–	–	–	–	–	–	–
10. CEQ Total	−0.091	0.166	0.19	−0.01	0.178	0.154	0.104	0.276**	0.166	–	–	–	–	–	–	–
11. CEQ Fear	−0.237**	−0.143	−0.121	−0.242	−0.047	−0.64	0.006	0.069	0	0.710**	–	–	–	–	–	–
12. CEQ Grief	−0.129	0.315**	0.265**	0.085	0.234*	0.252*	0.097	0.325**	0.148	0.847**	0.443**	–	–	–	–	–
13. CEQ Physical Distress	−0.056	−0.054	0.057	−0.103	−0.012	−0.063	0.078	0.094	0.006	0.769**	0.595**	0.505**	–	–	–	–
14. CEQ Insanity	−0.125	0.045	0.062	−0.108	0.076	0.039	0.034	0.163	0.11	0.630**	0.596**	0.399**	0.488**	–	–	–
15. CEQ Isolation	−0.144	0.033	0.084	−0.114	0.131	0.107	0.007	0.226*	0.19	0.698**	0.489**	0.601**	0.425**	0.478**	–	–
16. CEQ Death	−0.08	0.12	0.185	0.023	0.260*	0.202*	0.14	0.328*	0.221*	0.541**	0.382**	0.448**	0.297**	0.539**	0.373**	–
17. CEQ Paranoia	−0.357**	−0.13	−0.194	−0.184	0.109	0.142	−0.014	0.251*	−0.074	0.403**	0.402**	0.374**	0.305**	0.290**	0.342**	0.267**

Correlation values marked with ** indicate significance at *p* < 0.001 level. Correlation values marked with * indicate significance at *p* < 0.05 level. Dashes (−) indicate that there is no correlation calculated.

In the regression analysis, while controlling for age and gender, aesthetic experience was a strong positive predictor of emotional breakthroughs (*F*(3,92) = 10.20, *p* < 0.001)(*β* = 0.424), explaining 23% of the variance (Adjusted R^2^ = 0.225). Similarly, for mystical experiences, the regression model was significant (*F*(3,92) = 14.30, *p* < 0.001), with aesthetic experience as a strong positive predictor (*β* = 0.54, *p* < 0.001), explaining 30% of the variance in MEQ (Adjusted R^2^: 0.296). Importantly, for psychological insight and related behavioral change, the regression model was significant (*F*(3,92) = 11.40, *p* < 0.001; *F*(3,92) = 18.80, *p* < 0.001), with aesthetic experience again being a significant predictor (*β* = 0.51, *p* < 0.001; *β* = 0.61, *p* < 0.001) explaining 25 and 36% of the variance, respectively, (Adjusted R^2^: 0.247; Adjusted R^2^: 0.360). Age and gender were not significant predictors in any model.

### Association of aesthetic experience with negative experiences

In contrast to the positive experiences and in accordance with our hypothesis, aesthetic experience was negatively correlated with negative outcomes. Notably, a statistically significant negative relationship was found between aesthetic experience and the experience of fear (CEQ Fear) (*r*(94) = −0.24, *p* < 0.05), and the experience of paranoia (CEQ Paranoia) (*r*(94) = −0.36, *p* < 0.001), suggesting that a more aesthetic experience was associated with fewer challenging experiences in these factors. However, CEQ Total itself was not significantly associated with aesthetic experience.

To further investigate this relationship, regression models were applied to assess the impact of aesthetic experience on negative outcomes, controlling for age and gender. For the CEQ Total, the regression model was significant (*F*(3,92) = 9.16, *p* < 0.001). Both aesthetic experience (*β* = −0.45, *p* < 0.001) and age (*β* = −0.32, *p* = 0.002) were significant negative predictors, indicating that older individuals and those with more aesthetic experiences tend to report less fear; a finding consistent with previous research on older adults (Studerus et al., 2012; Kopra et al., 2022; Ko et al., 2023).

### Association of aesthetic experience with outcomes

Building upon the relationship to acute experiences, we evaluated several outcomes directly. Spearman correlations revealed that anxiety (*r*(94) = 0.35, *p* < 0.001), depression (*r*(94) = 0.40, *p* < 0.001), sleep quality (*r*(94) = 0.39, *p* < 0.001), alcohol use (*r*(94) = 0.21, *p* < 0.05) and quality of life (*r*(94) = 0.41, *p* < 0.001) exhibited significant positive correlations with aesthetic experience. In contrast, pain (*r*(94) = 0.15, *p* = 0.14) and opioid use (*r*(94) = 0.16, *p* = 0.14) did not demonstrate significant correlations.

To assess whether aesthetic experience significantly predicted psychological outcomes, we conducted linear regression analyses for each outcome variable, controlling for age and gender. Aesthetic experience was a significant predictor of several psychological outcomes. Specifically, higher aesthetic experience scores remained associated with greater anxiety (*β* = 0.24, *p* < 0.001), depression (*β* = 0.27, *p* < 0.001), alcohol use (*β* = 0.13, *p* < 0.05), improved sleep quality (*β* = 0.15, *p* < 0.01) and overall quality of life (*β* = 0.26, *p* < 0.001).

**Figure 2:**
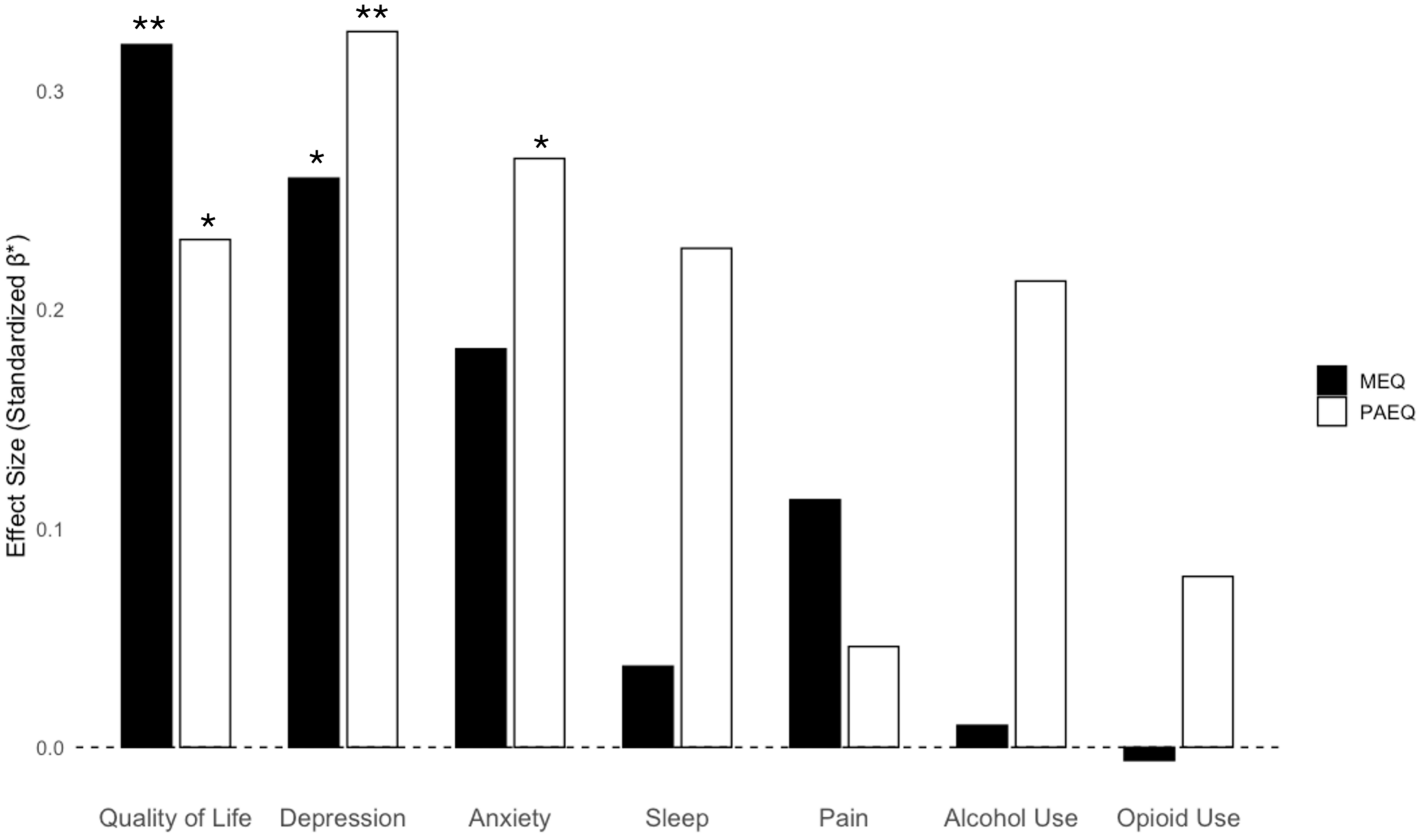
Standardized beta coefficients (β*) representing the predictive effects of aesthetic experience (PAEQ-S) and mystical experience (MEQ) on behavioral and psychological outcomes. Linear models were estimated for each outcome, with both PAEQ-S and MEQ included as predictors. Beta coefficients are standardized, allowing for direct comparison of effect sizes across predictors. A dashed line at zero represents no effect. Positive values reflect greater levels of the outcome associated with higher aesthetic or mystical experience scores, after adjusting for the other predictor. The results demonstrate that aesthetic experience has a significant positive effect on quality of life, depression, and anxiety, while effects on sleep quality, pain, and substance use were not significant. *p < 0.05, **p < 0.01.

Finally, MEQ Total score was included as a covariate to control for its potential qualitative overlap with aesthetic experience. After controlling for mystical experience, the effect of aesthetic experience remained significant for quality of life (*β* = 0.14, *p* < 0.05), depression (*β* = 0.19, *p* < 0.01), and anxiety (*β* = 0.16, *p* < 0.05), while sleep quality (*β* = 0.11, *p* = 0.07) and alcohol use (*β* = 0.10, *p* = 0.09) approached significance. In these cases, the effect of PAEQ-S was comparable to MEQ’s effect on outcomes, particularly for depression and anxiety (see Figure 2).

## Discussion

The present study adds to our understanding of how psychedelic experiences may contribute to positive psychological outcomes. While previous research has emphasized the significance of mystical and emotional breakthroughs (Barrett et al., 2015; Griffiths et al., 2018; Roseman et al., 2019), less attention has been given to the role of aesthetic components. Our findings suggest that psychedelic induced aesthetic enhancements are more than mere perceptual oddities, they appear to promote emotional engagement and cognitive shifts that may lead to enduring psychological benefits.

The correlation between aesthetic experience and psychological insight suggests that aesthetics may play a meaningful role in the therapeutic effects of psychedelics. The significant associations among mystical experiences, emotional breakthroughs, and aesthetic experiences further support the idea that, while these constructs are distinct, they contribute to overlapping psychological processes that are relevant for therapeutic outcomes. Notably, aesthetic experience was significantly correlated with improvements in psychological well-being. We found positive associations with improved quality of life, anxiety, and depression, even when controlling for potential confounders. These findings suggest that aesthetic engagement during psychedelic experiences may facilitate emotional and cognitive shifts, directly or indirectly impacting well-being. Future research utilizing pathway analysis or structural equation modeling (SEM) could help determine these relationships, clarifying the mechanisms through which aesthetic experience contributes to psychological effects and refining predictive models for therapeutic efficacy.

Another important finding is the relationship between challenging experiences, as measured by the CEQ, and aesthetic quality. Challenging experiences, such as fear and paranoia, are negatively correlated with positive psychological outcomes (Barrett et al., 2016). Likewise, we found that aesthetic experiences are inversely related to challenging experiences, indicating that when participants reported lower aesthetic quality, they were more likely to also encounter difficult emotional states. This relationship is particularly relevant for psychedelic-assisted therapy (PAT), as it suggests that enhancing the aesthetic environment could mitigate the intensity of challenging emotions, potentially leading to better therapeutic outcomes. Better understanding this dynamic could help optimize therapeutic protocols to create settings that promote positive emotional engagement while reducing the likelihood of adverse events.

Aesthetic experience is an emergent phenomenon arising from the integration of sensory-motor, emotion-valuation, and meaning-knowledge neural systems (Chatterjee and Vartanian, 2014), and it is studied empirically within the field of neuroaesthetics (Pearce et al., 2016). This framework suggests that aesthetic experience extends beyond passive sensory evaluation, engaging deeper affective and semantic processes. Notably, there is substantial evidence indicating that the reflective component of aesthetic experience is linked to the dynamics of DMN activity (Vessel et al., 2012, 2013, 2019; Belfi et al., 2019). A compelling parallel exists in the psychedelic literature, where DMN activity is consistently implicated, particularly in relation to the phenomenon of ‘ego death,’ which involves a dissolution of self-boundaries and heightened integration across neural systems (Carhart-Harris and Friston, 2010; Nour et al., 2016; Stoliker et al., 2022). This parallel, along with our data, presents a promising vein of research for future investigation utilizing advanced neuroimaging techniques.

Our findings, though cross-sectional, offer additional perspective on the mechanisms through which psychedelics exert their therapeutic effects. Future research could investigate whether manipulating the aesthetic environment during psychedelic sessions—such as through visual art, music, or immersive settings—might enhance the therapeutic process. Furthermore, longitudinal studies would be valuable in determining whether the lasting psychological benefits attributed to aesthetic experiences are maintained over time and whether specific aesthetic qualities are more effective than others in aiding psychological growth. Relatedly, (Aday et al., 2024) found that participants in a naturalistic ayahuasca study exhibited increased levels of aesthetic appreciation at both the 1-week and 1-month follow-ups.

## Limitations

This study has several limitations. First, its cross-sectional design prevents causal inference between psychedelic-induced aesthetic experiences and psychological outcomes. While significant associations were observed, it remains unclear whether enhanced aesthetics improve psychological well-being or if individuals with greater psychological openness report heightened aesthetic experiences. Longitudinal studies are needed to determine directionality.

Second, reliance on self-reported data introduces biases, including recall bias, social desirability, and expectancy effects. Participants may have exaggerated or minimized aspects of their experiences, influenced by cultural or personal expectations. Future research should integrate objective measures, such as psychophysiological responses or real-time experience sampling, to complement self-report data.

Third, the sample size (*n* = 96) was relatively small and recruited via social media and snowball sampling, introducing selection bias. Participants may have been more positively inclined toward psychedelics or had particularly salient aesthetic experiences. Additionally, the demographic composition limits generalizability. Future studies should recruit larger, more diverse samples to assess whether these effects extend across populations.

Fourth, the single-item measure of aesthetic experience (PAEQ-S), while practical, lacks granularity. The full 21-item PAEQ was excluded due to lack of prior validation, leading to a broad but potentially oversimplified assessment. This is particularly relevant for negative aesthetic experiences, which may differ qualitatively from positive ones. Additionally, single-item measures introduce ambiguity, as a “strongly disagree” response could reflect either a neutral or aversive experience. A forthcoming study will refine and validate the full PAEQ with a larger dataset.

Finally, while our analyses controlled for key demographic factors, unmeasured confounders remain. Variables such as set and setting, prior psychedelic use, dose, and pre-existing mental health conditions were not explicitly accounted for but may have influenced outcomes. Future research should systematically address these factors. Despite these limitations, this study offers a novel exploration of psychedelic-induced aesthetic experiences and their psychological implications. Addressing these methodological challenges will help clarify underlying mechanisms and therapeutic potential.

## Conclusion

This study demonstrates that the aesthetic quality of psychedelic experiences may be a crucial factor in predicting positive psychological outcomes, corroborating claims that subjective experience is necessary for psychedelic-induced therapeutic effects. By enhancing perceptual and emotional sensitivity, psychedelics create an aesthetic experience that may contribute to psychological insight and emotional breakthroughs, offering a novel mechanism for understanding their therapeutic potential. Understanding how these experiences operate provides a more comprehensive framework for the use of psychedelics in therapeutic settings, potentially informing clinical protocols that integrate aesthetic elements to maximize psychological benefit.

## Data Availability

The raw data supporting the conclusions of this article will be made available by the authors, without undue reservation.
