# Supplementary material for: Aesthetic quality of psychedelic experience is linked to insight and psychological outcomes
Source: Front Psychol. 2025 May 15;16:1533055. doi: 10.3389/fpsyg.2025.1533055 (PMC12119600; doi:10.3389/fpsyg.2025.1533055)
Supplement: Supplementary file 1 [file Data_Sheet_1.PDF]

Psychedelic Aesthetic Experience

Please rate how much you agree or disagree with the following statements regarding your most typical psychedelic event. Answer each question according to your feelings, thoughts, and experiences at the time of the event.

99% Progress

1) The visual patterns I experienced were vivid.
Strongly Disagree — Strongly Agree
(Place a mark on the scale above)

2) The visual patterns I experienced were complex.
Strongly Disagree — Strongly Agree
(Place a mark on the scale above)

3) The visual patterns I experienced were symmetrical.
Strongly Disagree — Strongly Agree
(Place a mark on the scale above)

4) The visual patterns I experienced were geometrical.
Strongly Disagree — Strongly Agree
(Place a mark on the scale above)

5) The visual patterns I experienced were smooth.
Strongly Disagree — Strongly Agree
(Place a mark on the scale above)

6) I experienced changes in color perception.
Strongly Disagree — Strongly Agree
(Place a mark on the scale above)

7) I experienced visions of objects, places, or entities.
Strongly Disagree — Strongly Agree
(Place a mark on the scale above)

8) My thought patterns were changed.
Strongly Disagree — Strongly Agree
(Place a mark on the scale above)

9) I experienced pareidolia (the tendency to see a specific, often meaningful image in a random or unclear pattern).
Strongly Disagree — Strongly Agree
(Place a mark on the scale above)

10) I gained new insights.
Strongly Disagree — Strongly Agree
(Place a mark on the scale above)

11) I saw the experience as an extension of myself.
Strongly Disagree — Strongly Agree
(Place a mark on the scale above)

12) I lost track of time.
Strongly Disagree — Strongly Agree
(Place a mark on the scale above)

13) I lost track of myself.
Strongly Disagree — Strongly Agree
(Place a mark on the scale above)

14) The experience was unique.
Strongly Disagree — Strongly Agree
(Place a mark on the scale above)

15) I experienced a heightened sense of beauty.
Strongly Disagree — Strongly Agree
(Place a mark on the scale above)

16) I experienced a heightened sense of ugliness.
Strongly Disagree — Strongly Agree
(Place a mark on the scale above)

17) I experienced a wide range of emotions that changed as I progressed through the experience.
Strongly Disagree — Strongly Agree
(Place a mark on the scale above)

18) I felt moved.
Strongly Disagree — Strongly Agree
(Place a mark on the scale above)

19) I experienced a physical reaction (changes in touch, taste, smell, temperature)
Strongly Disagree — Strongly Agree
(Place a mark on the scale above)

20) I was more creative.
Strongly Disagree — Strongly Agree
(Place a mark on the scale above)

21) The experience was aesthetically pleasing.
Strongly Disagree — Strongly Agree
(Place a mark on the scale above)
